# MicroRNAs and mesenchymal stem cells: hope for pulmonary hypertension

**DOI:** 10.5935/1678-9741.20150033

**Published:** 2015

**Authors:** Zhaowei Zhu, Zhenfei Fang, Xinqun Hu, Shenghua Zhou

**Affiliations:** 1 The Second Xiangya Hospital, Central South University, Huan Province, P.R. China.; 2 Department of Cardiology, Second Xiangya Hospital of Central South University, Huan Province, P.R. China.

**Keywords:** Hypertension, Pulmonary, MicroRNAs, Mesenchymal Stem Cell Transplantation

## Abstract

Pulmonary hypertension is a devastating and refractory disease and there is no cure for this disease. Recently, microRNAs and mesenchymal stem cells emerged as novel methods to treat pulmonary hypertension. More than 20 kinds of microRNAs may participate in the process of pulmonary hypertension. It seems microRNAs or mesenchymal stem cells can ameliorate some symptoms of pulmonary hypertension in animals and even improve heart and lung function during pulmonary hypertension. Nevertheless, the relationship between mesenchymal stem cells, microRNAs and pulmonary hypertension is not clear. And the mechanisms underlying their function still need to be investigated. In this study we review the recent findings in mesenchymal stem cells - and microRNAs-based pulmonary hypertension treatment, focusing on the potential role of microRNAs regulated mesenchymal stem cells in pulmonary hypertension and the role of exosomes between mesenchymal stem cells and pulmonary hypertension.

## Abstract (pt)

A hipertensão pulmonar é uma doença devastadora e refratária, para a qual não existe cura. Recentemente, microRNAs e células-tronco mesenquimais emergiram como novos métodos para tratar a hipertensão pulmonar. Mais de 20 tipos de microRNAs podem participar no processo de hipertensão pulmonar. Ao que parece, microRNAs ou células-tronco mesenquimais podem atenuar alguns sintomas de hipertensão pulmonar em animais de e até mesmo melhorar a função cardíaca e do pulmão durante a hipertensão pulmonar. No entanto, a relação entre células-tronco mesenquimais, microRNAs e hipertensão pulmonar não é clara. E os mecanismos subjacentes a sua função ainda precisam ser investigados. Neste estudo, revisamos as descobertas recentes no tratamento da hipertensão pulmonar baseado em células-tronco mesenquimais e microRNAs, enfocando o papel potencial dos microRNAs para regular as células-tronco mesenquimais na hipertensão pulmonar e o papel dos exossomos entre células-tronco mesenquimais e hipertensão pulmonar.

**Table:** 

**Abbreviations, acronyms & symbols**	
BMPR2	Bone morphogenetic protein receptor type II
BMSCs	Bone marrow stromal cells
CTEPH	Chronic thromboembolic pulmonary hypertension
HGF	Hepatocyte growth factor
IGF	Insulin-like growth factor
MSCs	Mesenchymal stem cells
PH	Pulmonary hypertension
RV	Right ventricle

## INTRODUCTION

Pulmonary hypertension (PH) is a devastating and refractory disease which is defined by a resting mean pulmonary artery pressure at or above 25 mmHg^[1]^. Untreated chronic PH can cause a hemodynamic and pathophysiological vicious cycle leading to right ventricle (RV) failure and despite modern treatments, the 3-year survival remains less than 60%^[2]^. Although currently there is no cure for this disease, treatment has been improved during the past decade, offering both relief from symptoms and prolonged survival. Recently, the regenerative method and gene therapy have been introduced to break the vicious cycle of PH. For example, transplantation of bone marrow-derived mesenchymal stem cells (MSCs) is emerging as a regenerative method to treat PH^[3,4]^. However, current evidence indicates that the efficacy of MSCs transplantation was unsatisfactory, due to the poor viability and massive death of the engrafted MSCs in the injured tissue. MicroRNAs are short endogenous, conserved, non-coding RNAs and important regulators involved in numerous facets of pathophysiologic processes. There is an obvious involvement of microRNAs in cell differentiation, neovascularization, apoptosis, and others. Nevertheless, the relationship between MSCs, microRNAs and PH is not clear. Here we review the recent findings in MSCs- and microRNAs-based PH treatment, focusing on the potential role of microRNAs regulated MSCs in PH.

## MSCS AND PH

MSCs are multipotent progenitor cells that were originally identified in the bone marrow stroma. MSCs have several favorable features for the transplantation therapy of pulmonary hypertension. Besides the ease of isolation and expansion in culture and their capacity to differentiate into multiple lineages, MSCs: have been shown to migrate to sites of injury; they have key interactions with the immune system and generate strong paracrine effects^[5]^. In addition, Firth et al.^[6]^ identified that a myofibroblast cell phenotype arising from transdifferentiation of differentiation of mesenchymal progenitor cells is predominant within endarterectomized tissues, contributing extensively to the vascular lesion/clot. These properties and findings make MSCs treatment a novel and promising approach for protection from and repair of PH. Recently, A number of animal studies taking use of monocrotaline or hypoxia induced animal model in pulmonary medicine have demonstrated that naive or gene-modified mesenchymal stem cells from bone marrow can ameliorate some of the symptoms of pulmonary hypertension. More interesting, both intratracheal and intravenous administration of MSCs can attenuate pulmonary hypertension in the aspects from endothelial dysfunction^[7]^, alveolar loss and lung inflammation^[8]^ even to ventricle remodeling^[7,9-11]^.

Further researches using gene-modified mesenchymal stem cells treatment also seem successful. Recent studies have found that eNOS^[12]^ or prostacyclin synthase^[13]^ or lung-specific HO-1^[14]^ modified MSCs can not only offer ameliorating effects on PH-related RV impairment but also improve the prognosis and even survival time in PH animals. Although haven't been applied to PH in clinic, all the studies above really provide us a hopeful prospect of MSCs transplantation therapy for PH.

However, the mechanisms of MSCs' therapeutic efficacy are still unclear. Although a robust protection against lung injury on MSCs treatment was observed in most of the above-mentioned animal models, only a small fraction of administered MSCs were detected in the wall of the pulmonary vessels^[15]^. This observation suggested that engraftment and direct tissue repair were not the sole mechanisms of MSC therapeutic function, and paracrine mechanisms were contemplated.

It is known that MSCs can be mobilized from the total pool of bone marrow stromal cells (BMSCs) when influenced by hypoxia or other injury factors^[16]^. After mobilization, it can localize into the injured tissue, and even few MSCs can fuse with cells from the host^[10]^. In addition to being mobilized into the circulation, MSCs have been shown to increase production of growth factors, such as VEGF, insulin-like growth factor (IGF), and hepatocyte growth factor (HGF), when under stress by TNF or hypoxia^[17,18]^. It is possible that transplanted MSCs may repair injured vascular endothelium by an action involving the release of factors that improve endothelial function or stimulate vascular growth in the injured lung^[19,20]^, which can be partly confirmed by the inhibition of lung inflammation after systemic delivery of MSCs-conditioned media^[8]^. So, mechanisms for this protection may be not limited to tissue repair, such as engraftment and differentiation of MSCs into specific lung cell types, but also include paracrine factors^[7,10,21]^. Considering the few numbers of MSCs located in injury tissue, MSCs paracrine signaling maybe a primary mechanism accounting for the beneficial effects of MSCs on responses to injury such as PH.

Among all the paracrine types of MSCs, exosomes, as mediators of cell-cell communication, provide a novel insight into the efficient role of MSCs in PH^[11]^. Exosomes are a kind of better-defined subclass of secreted membrane microvesicles, which are usually 30 to 100 nm in diameter. They have been isolated and characterized from various cell types, including MSCs. Recent study found MSCs-derived exosomes can exert a pleiotropic protective effect on the lung and inhibit pulmonary hypertension through suppression of hyperproliferative pathways, including STAT3-mediated signaling induced by hypoxia^[22]^.

## MICRORNAS AND PH

MicroRNAs (miRs) are small, non-coding RNAs regulating gene expression at the post-transcriptional level by mRNA degradation or translational repression^[23]^. The human genome has been estimated to contain up to 1000 miRNAs^[24]^. Many miRNAs exhibit a tissue-specific distribution and they appear to play a key role in cell function both under physiological and pathological conditions. Lots of *in vivo* and *in vitro* experiments related with functions of microRNAs in PH have emerged recent years. From animal experiments to clinical trials, microRNA expression profiles in PH have been revealed. A range of miRNAs are dysregulated in the lungs of rats exposed to chronic hypoxic and the monocrotaline model of PAH^[25]^. MiR-22, miR-30 and let-7f were down regulated, whereas miR-322 and miR-451 were up regulated significantly during the development of PH in both hypoxic and monocrotaline models. miR-21 and let-7a were significantly reduced only in monocrotalinetreated rats. miR-204 was consistently down regulated in pulmonary artery smooth muscle cells (PASMCs) from patients with PH and in cells from mice with PH^[26]^. Besides, circulating miR-150 and miR-26a levels are reduced in patients with poor survival in PH^[27,28]^. All the evidences above indicate a potential role of microRNAs in PH (Table 1).

**Table 1:** Summary of microRNAs which may play a potential role in PH.

microRNAs	Expression in PH	Research object	Function in PH	Mechanisms	References
MiR-22	reduced	animal	unknow	unknow	25
miR-30	reduced	animal	unknow	unknow	25
let-7f	reduced	animal	unknow	unknow	25
miR-21	reduced	animal and human	unknow	unknow	25
let-7a	reduced	animal	unknow	unknow	25
miR-150	reduced	human	protect	unknow	27
miR-204	reduced	animal and human	protect	target SHP2, inhibit PASMCs proliferation	26
miR-322	increased	animal	unknow	unknow	25
miR-451	increased	animal	unknow	unknow	25
miR-210	increased	animal	impair	Antiapoptotic effect in PASMCs	29
miR-21	increased	animal	impair	Enhanced the proliferation of human PASMCs	31,32
miR-759	increased	human	protect	Decrease the amount of total FGA mRNA via affecting the stability of FGA long isoform (aE) mRNA, which can contribute to CTEPH.	35
				
miR-17/92, 20a	increased	animal	impair	STAT3-miR-17/92-BMPR2 pathway	37,40
miR-145	incerased	animal and human	impair	Inhibit the BMPR2 function	38,39
miR-143	increased	animal and human	impair	Prevents down-regulation of KLF4 and activation of contractile genes by TGF-β or BMP4	38
miR-17	increased	animal	impair	up-regulation of p21	41
miR-206	decreased	animal	protect	down regulating Notch-3	43
miR-328	decreased	animal and human	protect	Inhibit L-type calcium channel-a1C expression	44
miR-26a	decreased	animal and human	unknow	unknow	28
miR-424	decreased	animal	protect	FGF2 pathways	42
miR-503	decreased	animal	protect	FGF2 pathways	42

As we all know, hypoxia is an important pathogenesis in PH. Inductions of microRNAs can be observed in SMCs cultured with hypoxia and in whole lungs of mice with chronic hypoxia-induced PH. miR-210 is the predominant miRNA induced by hypoxia, which has also been demonstrated by microarray analysis on human in hypoxic PASMCs and in whole lungs of hypoxic mice^[29]^. The induction of miR-210 is HIF-1α-dependent and triggers anti-apoptotic effects via directly targeting the transcription factor E2F3^[29]^. Previously study performed on pulmonary artery endothelial cells (PAECs) found that miR-210 can provide an adaptation to hypoxic conditions by targeting Iron-Sulfur Cluster Assembly Proteins 1/2 (ISCU)^[30]^.

MiR-21 can also be induced by hypoxia and overexpression of miR-21 enhanced the proliferation of human PASMCs in vitro and the expression of cell proliferation associated proteins, such as proliferating cell nuclear antigen, cyclin D1, and Bcl-xL, which indicates that miR-21 plays an important role in the pathogenesis of chronic hypoxia-induced pulmonary vascular remodeling^[31,32]^. Previous study showed that BMP-dependent signaling activation of miR-21 represses Rho-kinase activation in pulmonary artery endothelial cells, thus counteracting the Rho signaling in promoting pulmonary vascular pathology^[33]^. Besides, miR-21-null mice presented overexpression of RhoB and hyperaction of Rho-kinase activity accompanied by exaggerated manifestation of PH^[32]^.

Chronic thromboembolus is another leading cause of severe PH^[34]^. Chen et al.^[35]^ investigated the involvement of miR-759 in chronic thromboembolic pulmonary hypertension (CTEPH). CTEPH is characterized by persistent pulmonary embolism that increases pulmonary vascular resistance, resulting in pulmonary hypertension and subsequent right ventricular heart failure. The 3'UTR of FGA was found to interact with miR-759, and a 28-bp deletion polymorphism at this site was found to be more frequent in patients with CTEPH.

Further studies have been investigated to elucidate the concrete mechanisms of microRNAs participating in PH those years. As we all know, bone morphogenetic protein receptor type II (BMPR2), a receptor for the transforming growth factor (TGF-) b family, plays an important role both in endothelial and vascular smooth muscle cells and vascular remodeling of the pulmonary arterial circulation^[36]^. Several Studies have been designed to identify miRNAs that could inhibit the translation of BMPR-II, and members of the miRNA cluster 17/92 and miR-143, miR-145, miR-20a were identified as the potential regulators^[37-40]^. All these microRNAs inhibit the BMPR2 function, which were confirmed by experiments in either the patient vascular cells or the PH animal model. miR-145 and miR-143, two highly expressed miRNAs in SMCs, have been shown to play a pivotal role in the modulation of SMC phenotype. In particular, their expression is transcriptionally activated by both TGF-β and BMP4 and promotes a contractile phenotype in SMC by targeting the Kruppel-like factor-4 (KLF4)^[38]^.

Besides the research in pathogenesis and mechanisms, there are also investigations in therapy efficacy of microRNAs for PH in animal models. Pullamsetti et al.^[41]^ demonstrated that inhibition of miR-17 improves heart and lung function in experimental PH by interfering with lung vascular and right ventricular remodeling. The beneficial effects may be related to the up-regulation of p21. And recently, Kim et al.^[42]^ found that reconstitution of miR-424 and miR-503 can ameliorate pulmonary hypertension in experimental models through FGF2 pathways.

Although recent studies found that most PH-related microRNAs usually play a negative role in the pathogenesis process of PH, interestingly, there are still some microRNAs which can play a protective role in PH. miR-204 and miR-206 are two well researched microRNAs, both of which are down regulated in PASMCs from patients with PH or in cells from mice with PH. They all paticipate in the SMCs' proliferation and apoptosis and even differentiation. miR-204 was consistently down regulated in PASMCs from patients with PAH and in cells from mice with PAH^[26]^. miR-204 show a direct influence on PASMC function and delivery of miR-204 mimics to the lungs of mice with PAH significantly can reduce disease severity. miR-206 can alleviate PAH through down regulating Notch-3 expression, which is key a factor in PAH development^[43]^.

Besides, hypoxia produced a significant inhibition of miR-328 expression, which has been identified as a strong candidate responsible for hypoxic pulmonary vasoconstriction. Overexpressing miR-328 in the transgenic mice remarkably decreased the right ventricular systolic pressure and PA wall thickness under both normoxia and hypoxia. Through inhibiting L-type calcium channel-α1C expression the insulin growth factor 1 receptor, ultimately leading to apoptosis of pulmonary arterial smooth muscle cells^[44]^.

## POSSIBLE AND NOVEL LINK BETWEEN MSCS AND MIRNAS IN PH

It is well known that miRNAs have been implicated in many processes of stem cell functions, including cell proliferation, differentiation and apoptosis. Recent studies^[45]^ suggest that mesenchymal stem cells have discrete miRNA expression profiles that can account for the intrinsic stem cell properties of self-renewal and pluripotency. Through certain modified microRNAs, up or down regulation, there must be ways to enhance the viability of engrafted MSCs in the injured pulmonary tissue.

Exosomes have emerged as a novel media between kinds of cells. And exosomes based therapy has been confirmed by many researches. In consideration of its microRNAs-carried function, it is feasible to treat with PH by the microRNAs-carried exosomes secreted by MSCs. Recently, this hypothesis has been confirmed in a research that demonstrate MSCs can regulate neurite outgrowth by transfer of miR-133b to neural cells via exosomes^[46]^.

In a word, either mimicking or antagonizing microRNA actions, MSCs functions can be regulated by microRNAs to enhance the properties of cell differentiation or anti-apoptosis. Considering that microRNAs can be delivered by exosomes secreted by MSCs, it is likely that overexpression of special microRNAs like miR-204/206/328 in MSCs will hopefully enhance MSCs therapeutic efficacy for PH. So, microRNAs may be used as novel regulators in MSC-based therapy in PH and microRNAs-regulated MSCs transplantation may represent promising therapeutic strategy for PH patients in the future.

**Table:** 

**Authors’ roles & responsibilities**	
ZZ	Study conception and design
ZF	Study conception and design
XH	Analysis and/or interpretation of the data
SZ	Final approval of the manuscript
